# Alginate-Edible Coatings for Application on Wild Andean Blueberries (*Vaccinium meridionale* Swartz): Effect of the Addition of Nanofibrils Isolated from Cocoa By-Products

**DOI:** 10.3390/polym12040824

**Published:** 2020-04-05

**Authors:** Carolina Medina-Jaramillo, Carmen Quintero-Pimiento, Catalina Gómez-Hoyos, Robin Zuluaga-Gallego, Alex López-Córdoba

**Affiliations:** 1 Facultad Seccional Duitama, Escuela de Administración de Empresas Agropecuarias, Universidad Pedagógica y Tecnológica de Colombia, Carrera18 con Calle 22, Duitama 150461, Colombia; caromedina1986@gmail.com (C.M.-J.); rosaquintero885@gmail.com (C.Q.-P.); 2 Programa de Ingeniería en Nanotecnología, Universidad Pontificia Bolivariana, Circular 1° N° 70-01, Medellín 050031, Colombia; catalina.gomezh@upb.edu.co; 3 Facultad de Ingeniería Agroindustrial, Universidad Pontificia Bolivariana, Circular 1° N° 70-01, Medellín 050031, Colombia; robin.zuluaga@upb.edu.co

**Keywords:** edible films and coatings, biopolymers, nanocellulose, agro-food by-products, food packaging, wild blueberries

## Abstract

Edible coatings and films are appealing strategies for the postharvest management of blueberries. In the current work, alginate and alginate/cellulose nanofibril (CNF) edible coatings crosslinked with calcium chloride were developed for application on Andean blueberry (a promissory wild blueberry). Cocoa by-products were valorized through the isolation of their CNFs, and these were incorporated in the edible coatings. Edible coating formulations were based on blends of alginate (2% *w*/*v*), CNFs (0%, 0.1%, or 0.3%), glycerol, and water. In addition, stand-alone films were prepared, and their light and water vapor barrier properties were studied before applying the coating on the fruit surface. The results show that the addition of CNFs caused a significant decrease in the transparency and the water vapor permeability of the alginate films. After applying on the Andean blueberry fruits, the alginate and alginate/CNF coatings enhanced the appearance and the firmness of the fruits. Moreover, they significantly reduced the respiration rate and the water loss of the Andean blueberries throughout the 21 days of refrigerated storage. Alginate and alginate/CNFs coatings may be considered a useful alternative for the delay of the postharvest deterioration of Andean blueberries.

## 1. Introduction

Blueberries are widely consumed small red fruits with high economic value and several health benefits [1]. Highbush blueberries (*Vaccinium corymbosum*) are the most commercial blueberries. However, there are several other wild shrubs of the genus *Vaccinium* with similar or higher biological activity, but with few commercial exploitations, such as the *Vaccinium meridionale* Swartz (Andean blueberry). The latter grows in the Andean region of South America at 2300–3300 m above sea level (m.a.s.l.) [2]. 

Andean blueberry fruits are rich in bioactive compounds such as anthocyanins (e.g., cyanidin-3-galactoside and cyanidin-3-glucoside), flavonoids (e.g., quercetin glycosides), and phenolic acids (e.g., chlorogenic acid) [3]. It has been reported that Andean blueberry phytochemicals have several biological activities, including antioxidant, cardioprotective, antiproliferative, and anti-inflammatory properties [2]. Therefore, these fruits have a high potential of use as an antioxidant or functional ingredient in cosmetics, pharmaceutical, and agri-food applications [2]. However, Andean blueberry fruit decays quickly at ambient temperature after harvest and is susceptible to mechanical injury, water loss, and microbial attack during postharvest storage [4]. Moreover, Andean blueberries have tended to look less appealing than most commercial blueberries, such as Highbush blueberries. Storage at low temperature (0–2 °C) and high relative humidity (90%) is the current method commonly used for maintaining the quality of blueberries at an industrial scale. However, there is a rising interest in the use of new postharvest technologies such as edible coatings, ultraviolet irradiation, and active and smart packaging [5].

Alginates are a family of unbranched binary copolymers of (1─4)-linked β-d-mannuronic acid (M) and α-l-guluronic acid (G) residues of widely varying composition and sequential structure [6]. The food industry uses alginates for specific gelling, thickening, and stabilizing applications. Moreover, alginates are well known for their good film-forming properties and functionalities [7]. Although those alginate edible films are water-soluble, they can be turned insoluble through the cross-link with divalent or polyvalent cations such as Ca^2+^ [7]. 

Alginate-based edible coatings have been useful in maintaining the postharvest quality of blueberry, tomato, peach, sweet cherry, pineapples, and plums, among others [8,9,10,11]. Moreover, through the blend of alginate with food preservatives and/or other food biopolymers, it is possible to obtain active edible coatings with improved functional properties [9,12,13]. It has been reported that the addition of nano-reinforcement agents can improve the mechanical properties and the water vapor barrier of alginate films [14].

Cellulose has been reported as a suitable raw material for the formulation of films and edible coatings because it is an abundant, renewable, non-toxic, and cheap biopolymer [15,16]. In particular, the use of nanocellulose has gained a growing interest in the last years because of its high surface area and aspect ratio, absence of cytotoxic and genotoxic properties, and appealing physicochemical properties [17]. There are several sources of cellulose including algae (e.g., *Valonica ventricosa* and *Chaetamorpha melagonicum*), bacteria (e.g., members of *Acetobacterium* genus and others), marine animals like tunicates (e.g., *Microcosmus fulcatus*), wood, cotton, plants (e.g., hemp, flax, jute, and ramie), and agricultural by-products (e.g., wheat straw, banana rachis, and cocoa shell) [15,18,19]. 

Cocoa shells (*Theobroma cacao* L.) are a by-product of cocoa processing; they represent 12 wt.% of the raw material [20]. This by-product is underestimated and has been mainly used as fuel for boilers in the formulation of animal food and the manufacture of fertilizers [20]. Recently, some studies and patents have been developed, suggesting alternative applications for this material, as it contains very interesting compounds from a nutritional point of view, such as phenolic compounds, fibers, and a significant fat content with a lipid profile very similar to that of cocoa butter [21,22,23]. Moreover, considering its composition, it is also possible to promote a different approach so the cocoa shell can be used to produce nanostructures such as vegetable nanocellulose fibers [15], which can be used in applications like bionanocomposites for edible coatings, to improve the efficiency of the productive chocolate chain.

Several authors have proposed the preparation of value-added bionanocomposites through the blending of biodegradable polymers (e.g., polycaprolactone and starch) with cellulose nanofibrils from cocoa pod husk. It has been reported that the addition of cocoa cellulose nanofibrils (CNFs) in bioplastics allows materials with improved mechanical and barrier properties to be obtained [24,25]. However, to the best of our knowledge, there are few studies about the application of CNFs from cocoa shells in the formulation of edible films and coatings.

The objective of the current work was to evaluate the effect of alginate and alginate/CNF edible coatings on the physicochemical properties of wild Andean blueberries (*Vaccinium meridionale* Swartz) during refrigerated storage. Control and coated Andean blueberries were monitored during 21 days of storage in terms of their pH, titratable acidity (%), soluble solids content, respiration rate, firmness, weight loss, and fungal decay. The findings suggested that alginate and alginate/CNF edible coatings were suitable for the delay of the postharvest deterioration of Andean blueberries during the refrigerated storage.

## 2. Materials and Methods 

### 2.1. Materials

Cocoa shell of the main variety of cocoa beans (*Theobroma cacao* L.), cultivated in Colombia, was supplied by Compañía Nacional de Chocolates after the roasting process. This cocoa shell was ground using a Resch mill to pass a 1 mm screen (Mesh No. 18).

Andean blueberries (*Vaccinium meridionale* Swartz) at maturity stage 4 (100% purple) were obtained in Ráquira (Boyacá, Colombia) at 2150 m.a.s.l. The berries were examined previous to its use to separate fruits with physical, mechanical, or microbial damages. 

All chemicals used were of analytical grade. Sodium alginate was kindly donated by Saporiti (Buenos Aires, Argentina). Glycerol was purchased from J. T. Baker (Phillipsburg, New Jersey, USA). Sodium hydroxide and calcium chloride were purchased from Sigma Aldrich (St. Louis, MO, USA). 

### 2.2. Isolation of Cellulose Nanofibrils (CNF) from Cocoa Byproducts

Cocoa shell was chemically treated following a modification of the KOH-5 procedure developed by Zuluaga et al. [26] to remove non-cellulosic components. First, the cocoa shell was vigorously stirred at room temperature for 14 h with 5 wt.% KOH solution. Then, the insoluble residue was delignified with 1 wt.% NaClO_2_ at pH 5.0, and adjusted with 10 wt.% acetic acid, at 70 °C for 1 h. At each step of the different treatments, the insoluble residue was extensively washed with distilled water until the pH was neutral. Finally, the cellulosic material at a concentration of 2 wt.% was passed 30 times through grinder equipment (Masuko Sangyo, Supermasscolloider), according to the G30 procedure developed by Velásquez-Cock et al. [27].

### 2.3. Atomic Force Microscopy 

The morphology of cellulose nanofibrils from cocoa shell was studied using atomic force microscopy (AFM). Samples were imaged in tapping mode using a Flex AFM (Nanosurf) equipped with a multimode head and operated with a resonance frequency of 183 kHz. It used a cantilever of 125 mm in length and 5–10 nm in tip radius. Nanocellulose samples were diluted in distilled water and sonicated (Elma Elmansonic P) for 15 min at room temperature to achieve a good dispersion of the nanocellulose. Subsequently, a fine layer of the sample was deposited on mica by using a spin-coater for 2 min at 2000 rpm. Before morphological analysis, samples were stored in a vacuum desiccator for 3 days.

### 2.4. Preparation of Film/Coating Solutions

Film/coating solutions with and without cellulose nanofibrils from cocoa shell were prepared. Alginate solutions were made by dissolving sodium alginate powder (2% *w*/*v*) in distilled water and heating at 70 °C under constant stirring until the mixture became clear. Glycerol (30% *w*/*v* dry weight basis) was added as a plasticizer to the sodium alginate solution and stirred for 15 min. 

For the preparation of alginate/CNF blends, alginate solutions of two different concentrations of Cocoa CNFs (0.1% and 0.3% *w*/*v*) were added and homogenized at 20,000 rpm for 3 min using an Ultra Turrax T25 (IKA^®^ WERKE, Germany) homogenizer. The concentrations of sodium alginate, glycerol, and cocoa shell nanofibrils were chosen according to previous works [28].

All formulations were degassed using a vacuum pump and cooled to room temperature for later application on the fruits. 

### 2.5. Formation and Characterization of Edible Films

The film-forming solutions were dispensed into polypropylene plates and dried at 50 °C for 24 h. Then, dried films were peeled from the plates, submerged in a gelling bath of calcium chloride solution (1% *w*/*v*) for 30 min, cleaned with distilled water, and air-dried at room temperature. All films were conditioned at room temperature into desiccators containing a supersaturated solution of sodium bromide (RH ~ 57%) for 48 h prior to characterization studies. 

The thicknesses of the films were measured using an electronic digital caliper.

Water vapor permeability (WVP) tests were carried out at room temperature following the ASTM E96/ASTM E96M-16 method. Film samples were sealed over a circular opening of 4 × 10^−4^ m^2^ in a permeation cell, containing calcium chloride. Then, the cells were placed in desiccators conditioned with sodium chloride saturated solution (75% RH). Changes in the weight of the cell were recorded to the nearest 0.0001 g and plotted as a function of time, and the slope of each line was calculated by linear regression. WVP (g Pa^−1^ s^−1^ m^−1^) was calculated as follows:

WVP = [WVTR/P.RH] d,(1)

where WVTR is the water vapor transmission rate calculated as the ratio between the slope of the straight line (g/s) and the cell area (m^2^); P is the saturation vapor pressure of water (Pa); RH is the relative humidity in the desiccator, and d is the film thickness (m).

Film transparency was measured as reported by Piñeros-Hernandez, Medina-Jaramillo, López-Córdoba, & Goyanes, 2017 [29]. Films were cut into rectangles (50 mm × 10 mm) and placed on the inner side of a quartz spectrophotometer cell. The percent transmittance (% T) of light at 600 nm (T_600_) was measured using a UV-visible spectrophotometer (X-ma 1200 Human Corporation, Loughborough, UK), and the transparency was calculated as the ratio between logT_600_ and the thickness (mm) of each film.

### 2.6. Application of Edible Coatings

A total mass of 6 kg of Andean blueberries was randomly divided into four groups (control, alginate, alginate/CNFs 0.1%, alginate/CNFs 0.3%), each group containing 1.5 kg of fruit. The blueberries were dip-coated by immersion in the coating solutions for 90 s, drained of excess coating, submerged in a gelling bath of calcium chloride solution (1% *w*/*v*) for 30 min, cleaned with distilled water, and air-dried at room temperature. Control samples (without coating) were also prepared by immersion of fruits in distilled water and kept under the same storage conditions than the treated ones, for comparison.

The surface solid density (SSD) was estimated as an indicator of the coating’s average thickness as follows [30,31]:

SSD = [M_CA_. X_s_/A_s_], (2)

where SSD is the surface solid density (g/m^2^); M_CA_ is the mass of coating solution adhered to the fruit surface (g); X_S_ is the mass fraction of solid in the coating solution; and A_S_ is the surface area of Andean blueberries (m^2^). The average sample surface area (A_s_) was estimated by considering blueberry as a sphere. Samples were weighed before and after coating to determine the mass of coating solution adhered to the fruit surface (M_CA_). The non-coated sample was used as a control. 

### 2.7. Evaluation of Quality Attributes of Andean Blueberries along Storage 

The coated and uncoated Andean blueberries were packed in PET trays with perforated vents and stored for 21 days. Evaluations of quality attributes were performed at 1, 7, 14, and 21 days of refrigerated storage (4 °C and 90% RH). For every sampling time, three trays containing 125 g (~250 units) of Andean blueberries each were prepared.

#### 2.7.1. Respiration Rate

Respiration rate was measured as reported by Hasperué, Rodoni, Guardianelli, Chaves, & Martínez, 2016 [32]. Approximately 120 g of Andean blueberries were placed for 30 min at 25 °C inside hermetically sealed 2 L flasks. Then, the CO_2_ concentration was determined using an infrared analyzer (LabQuest^®^2 Model LQ2-LE, Beaverton, OR, USA), and results were expressed as the rate of respiration (CO_2_) in mg kg^−1^ s^−1^. 

#### 2.7.2. Weight Loss 

Weight loss of Andean blueberries during storage was determined by weighing all fruit trays at the beginning of the storage and every day of analysis. The weight loss (% *W*) was calculated with the following equation:

(3) $$\%\;W=\left(\frac{m_{0}-m_{f}}{m_{0}}\right)\times 100,$$

where m_f_ is the weight at each time and m_0_ the initial weight of each sample.

#### 2.7.3. Soluble Solids Content, pH, and Titratable Acidity (%)

The soluble solids content was measured in the fruit juice using an Atago refractometer model PR 101 (Atago CO., Tokyo, Japan) and expressed as Brix (AOAC 932.12). Fruit samples were crushed using a blender and filtered through filter paper to obtain the fruit juice. 

The pH of the fruit samples was assessed using a digital pH meter (Oakton Instruments, Vernon Hills, IL, USA) (AOAC 981.12).

Titratable acidity (%) was determined by titration with 0.1 N NaOH up to pH 8.2, using 0.5 g of sample in 10 mL of distilled water (AOAC 942.15). The results were expressed in citric acid percentage. 

#### 2.7.4. Firmness Analysis

Firmness was determined using a digital Force Gauge PCE-FM200 (Southampton, UK) equipped with a 6 mm diameter stainless steel probe. Firmness was defined as the maximum force to disrupt the tissue at the penetration time used (5 s) [33]. The results were expressed as an average of at least five measurements.

### 2.8. Statistical Analysis

The statistical analysis was performed using Minitab v. 16 statistical software (State College, PA, USA). Analysis of variance (ANOVA) and Tukey’s pairwise comparisons were carried out using a level of 95% confidence. The experiments were performed at least in triplicate, and the data were reported as mean ± standard deviation.

## 3. Results and Discussion

### 3.1. Characterization of Cellulose Nanofibrils (CNFs) from Cocoa Byproducts

**Figure 1:**
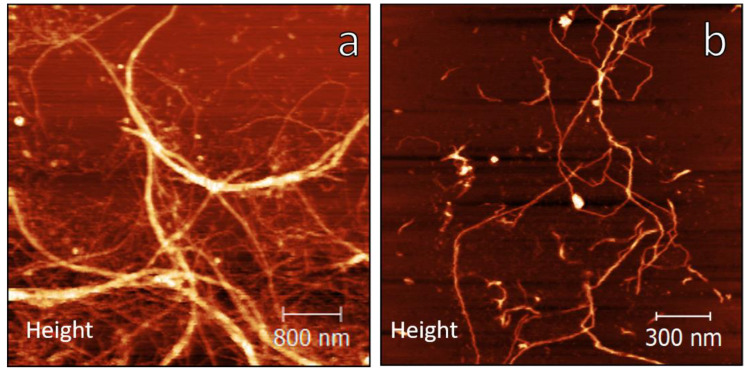
AFM height images of cocoa shell nanocellulose for 5 × 5 μm (**a**), and 2 × 2 μm (**b**) areas.

Figure 1a shows some bundles with diameters around 300 nm and some individual CNFs. The morphology of individual CNFs is evidenced more clearly in Figure 1b, where nanofibers with diameters between 15 and 50 nm and several microns in length are observed. Therefore, AFM images presented in Figure 1a,b reveal that the cellulosic material isolated from cocoa shell has a fibrillar morphology and forms an entangled network of high-aspect materials, which makes it a suitable material to develop edible coatings and films with barrier properties and prevent the gaseous exchange.

### 3.2. Film Characterization

Table 1 shows the thickness, transparency, and water vapor permeability of the alginate films with and without cellulose nanofibrils from cocoa shell. All films were clear enough to be used as see-through packaging. However, alginate/CNF 0.1% and alginate/CNF 0.3% films showed a decrease in the transparency of 27% and 40%, respectively, in comparison to the alginate ones. Martins et al. [34] also reported a higher opacity in starch/CNF films as more CNFs were added to the filmogenic matrix, and it was attributed to the dispersion of light by nano-sized fibrils.

In addition, the addition of cocoa shell nanofibrils caused a decrease in the water vapor permeability of the films of 65%, compared to the films without nanofibrils, regardless of the concentration used (0.1% or 0.3%). This behavior could be attributed to the nanofibrils being able to act as a filler, causing a tortuous pathway that limits water vapor transmission through the alginate matrix. These results are in agreement with those reported in the literature [34].

### 3.3. Behavior of Andean Blueberry Quality Parameters during Storage

**Figure 2:**
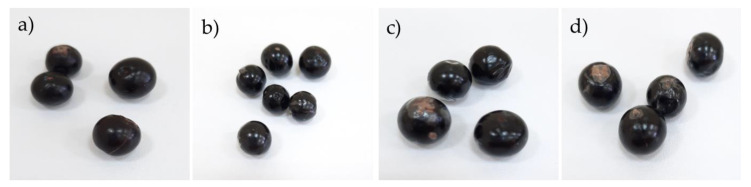
Images of Andean blueberries with and without coatings: (**a**) Control; (**b**) alginate; (**c**) alginate/cellulose nanofibrils (CNFs) 0.1%; and (**d**) alginate/CNFs 0.3%.

Figure 2 shows the appearance of Andean blueberries with and without coatings. It can be seen that the coated fruits were brighter than the uncoated ones. This behavior was attributed to the smoother surface of the coated fruit, compared to the fruit skin, and it caused a greater reflection of visible light [35]. The brightness is an important quality perceived attributed to blueberries because consumers commonly associate it with a fresh-like appearance [36].

Besides, the surface solid density (SSD) was estimated as an indicator of the applied coating’s thickness. It was observed that the addition of CNFs did not cause important changes in the SSD of the fruits, obtaining values of 0.52, 0.54, and 0.60 g.m^−2^ for the alginate, alginate/CNFs 0.1%, and alginate/CNFs 0.3% systems, respectively. SSD values between 1.0 and 1.85 g.m^−2^ were reported by Falcó et al. [31] for blueberries (*Vaccinium corymbosum*) coated with edible coatings based on carrageenan (κ−, ι−, and λ).

**Figure 3:**
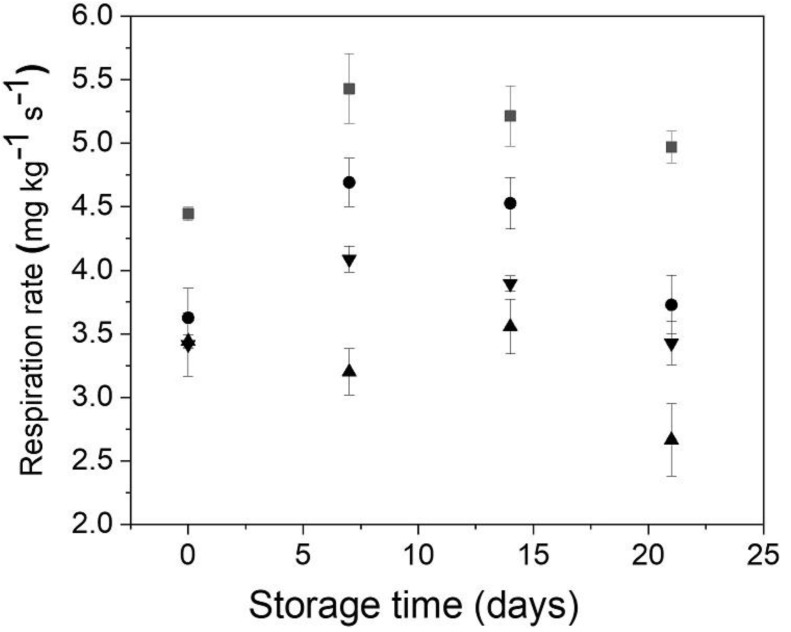
Behavior of the Andean blueberry respiration rate during storage. Control (■), alginate (●), alginate/CNFs 0.1% (▲), alginate/CNFs 0.3% (▼).

The changes in the respiration rate of Andean blueberries with and without edible coatings during storage are shown in Figure 3. All edible coatings caused a significant decrease in the respiration rate of the Andean blueberries. It has been well documented that alginate edible coatings are suitable to work as a barrier to oxygen, retarding the respiration rate, and also browning reactions [9,37,38]. At the end of the storage, the control fruits increased their respiration rate, while the fruits coated with alginate and alginate/CNFs 0.3% showed a similar respiration rate than at the initial time. Unlike the other samples, the fruits coated with alginate/CNFs 0.1% showed a significant decrease in their respiration rate at the end of the storage. It has been reported that CNFs with a high aspect ratio can turn and entangle into a network structure, leading to a tortuous diffusion path, which prevents the gaseous exchange [39,40]. However, above a critical concentration, CNFs tend to form agglomerate held together by hydrogen bonding [34].

**Figure 4:**
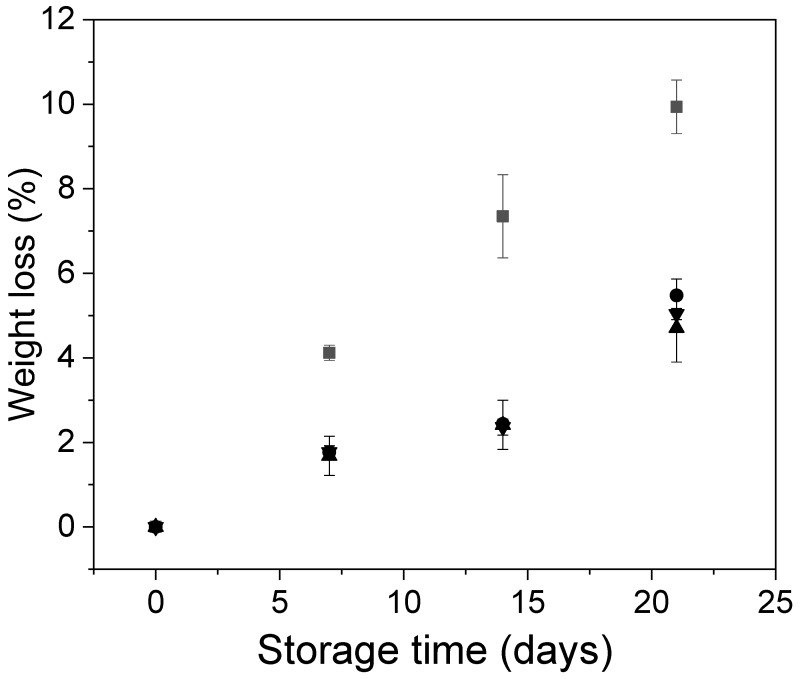
Behavior of the Andean blueberry weight loss during storage. Control (■), alginate (●), alginate/CNFs 0.1% (▲), alginate/CNFs 0.3% (▼).

Weight loss is a primary factor in blueberry deterioration [41]. It is well known that the loss of water in fresh fruits is mainly associated with respiration and transpiration through the skin, and it causes sensory quality loss generally because of the fruit shrinkage. Here, all systems showed a gradual increase in weight loss during storage (Figure 4). However, in the case of the coated fruits, the weight loss was significantly lower than in the control ones, regardless of the presence of cocoa shell nanofibrils. At the end of storage, control fruits and coated fruits showed water loss around 10% and 5%, respectively (Figure 4). It has been suggested that postharvest treatments that restrict blueberry water loss to below 8% are likely to be beneficial in retaining acceptable blueberry freshness and firmness [4,41]. The ability to reduce weight loss of alginate coatings has been demonstrated in several fruits, including pears [42] and plums [9]. 

**Figure 5:**
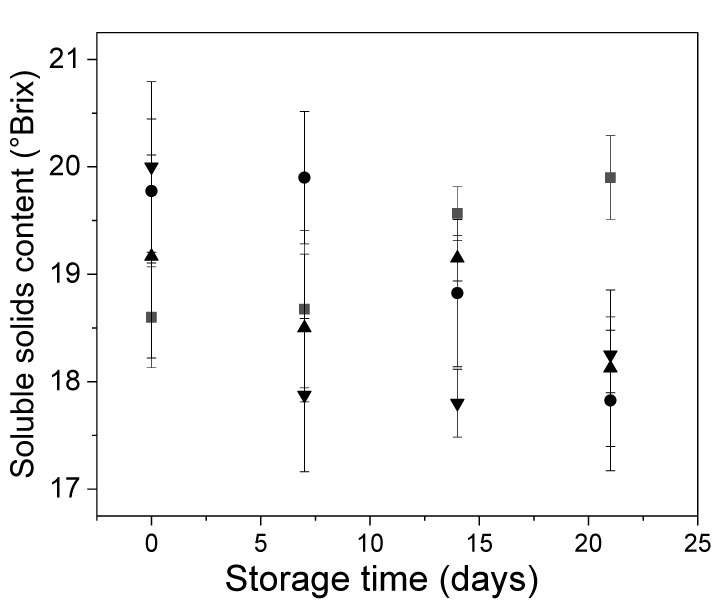
Behavior of the Andean blueberry soluble solids content during storage. Control (■), alginate (●), alginate/CNFs 0.1% (▲), alginate/CNFs 0.3% (▼).

**Figure 6:**
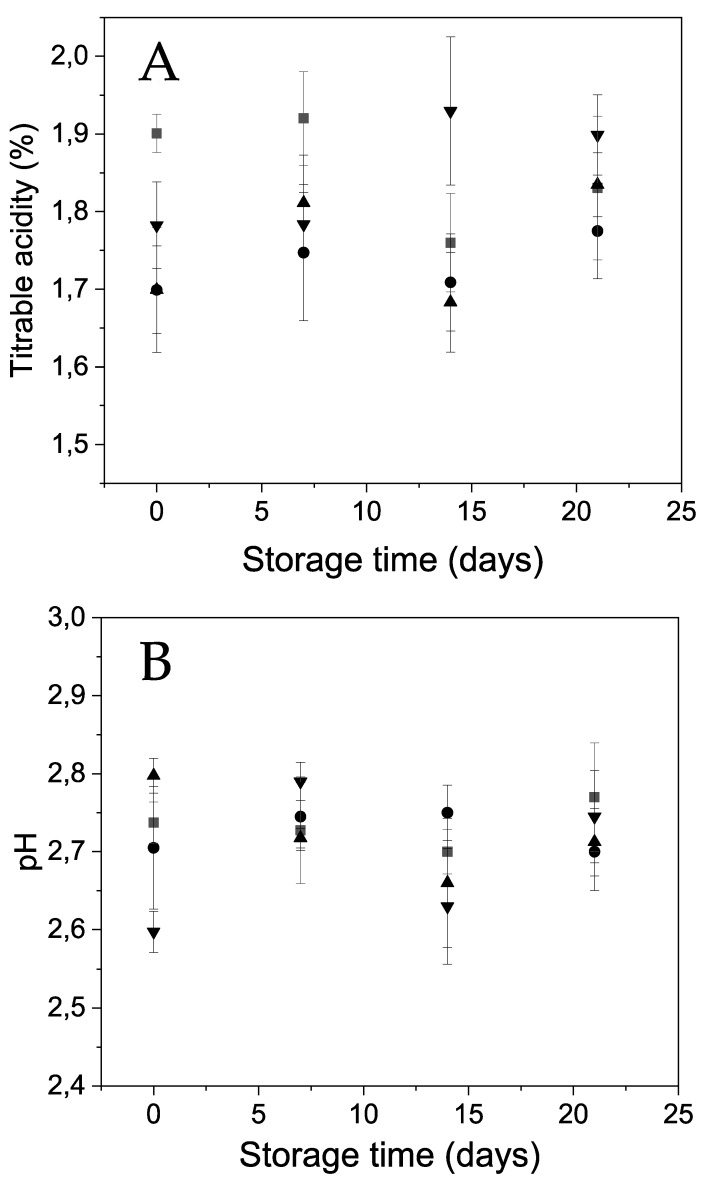
Changes in Andean blueberry (**A**) titratable acidity (%) and (**B**) pH during storage. Control (■), alginate (●), alginate/CNFs 0.1% (▲), alginate/CNFs 0.3% (▼).

The changes in Andean blueberry soluble solids content, titratable acidity, and pH are shown in Figure 5 and Figure 6. Control fruits showed a slight increase in their soluble solids content during storage (Figure 5). This behavior was probably due to both the water loss and the cell wall degradation caused by the fruit ripening. In the case of the coated samples, it was expected that the water loss caused an increase in the soluble solids contents. Therefore, it can be hypothesized that the edible coatings were able to prevent the cell wall degradation, avoiding the increase in the soluble solids content of the coated samples.

Concerning the pH and the titratable acidity (%), no relevant differences were detected among the uncoated and the differently coated samples neither at the initial time nor during the storage period (Figure 6).

**Figure 7:**
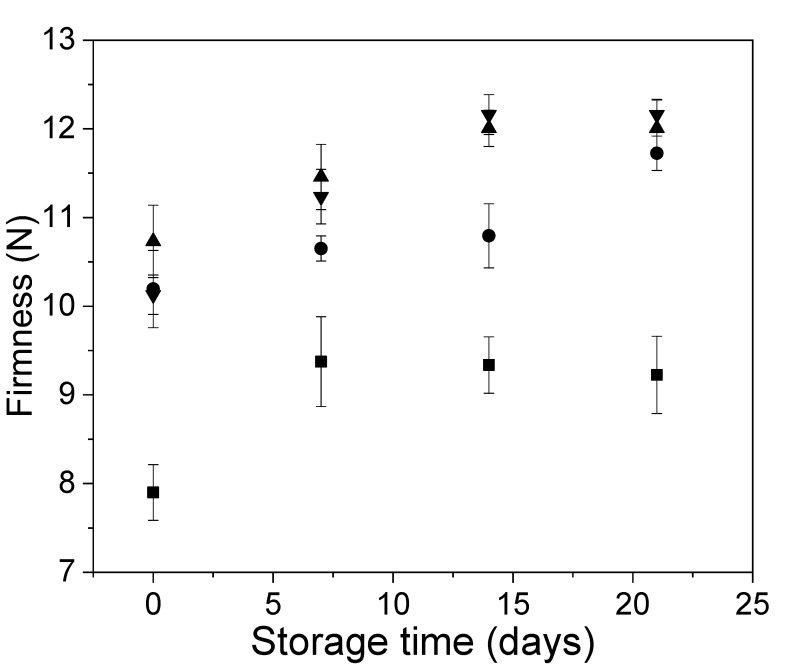
Changes in Andean blueberry firmness during storage. Control (■), alginate (●), alginate/CNFs 0.1% (▲), alginate/CNFs 0.3% (▼).

The behavior of the firmness of the Andean blueberries with and without edible coatings during storage is shown in Figure 7. All the coated fruits showed significantly higher firmness than the uncoated fruit throughout the storage. This behavior could be attributed to both the presence of a coating that provides structural rigidity to the surface of the fruit and to the use of calcium ions as a cross-linking agent, which could act as a textural enhancer, minimizing the softening [10,43]. In addition, the increase in the firmness of the samples throughout the storage could be associated with the increase in the water loss.

At the end of the storage, the firmness of coated fruits was 25% higher than that the control ones.

At the beginning of storage, the fruits with alginate/CNF 0.1% edible coatings showed higher firmness than the other systems. This fact could be due to the good dispersion of Cocoa shell nanofibrils within the alginate matrix when it was used at lower concentrations. Then, at 7 and 15 days of storage, the two coatings added of Cocoa shell nanofibrils provided higher firmness to the Andean blueberries. However, at the end of the storage, the firmness of the fruits with alginate and alginate/CNFs was similar.

## 4. Conclusions

Agro-food byproducts such as cocoa shell show promise as a precursor of cellulose nanofibrils than can be used in the formulation of edible films and coatings for their potential application in the preservation of foods such as Andean blueberries. 

Cellulose nanofibrils with diameters between 15 and 50 nm were successfully extracted from cocoa shells through chemical and mechanical treatment. When these cellulose nanofibrils were incorporated within calcium alginate matrices, they provoked a significant decrease in the water vapor permeability and the transparency of the films. Moreover, the alginate edible coatings were able to decrease the respiration rate and weight loss and to improve the firmness of Andean blueberries, regardless of the presence of cellulose nanofibrils. Therefore, it can be concluded that alginate and alginate/cellulose nanofibrils coatings were useful in the delay of the postharvest deterioration of Andean blueberries when compared to the fruits without coatings.

## Figures and Tables

**Table 1:** Thickness, water vapor permeability, and transparency of the alginate films with and without cellulose nanofibrils from cocoa shell.

Sample	Thickness (mm)	Transparency (%)	Water vapor permeability (g s^−1^ m^−1^ Pa^−1^ × 10^−10^)
Alginate	0.23 ± 0.01 ^a^	8.1 ± 0.4 ^a^	2.9 × 10^−9^ ± 0.4 ^a^
Alginate/CNFs 0.1%	0.24 ± 0.02 ^a^	5.9 ± 0. 2 ^b^	1.1 × 10^−9^ ± 0.1 ^b^
Alginate/CNFs 0.3%	0.22 ± 0.01 ^a^	4.8 ± 0.3 ^c^	0.8 × 10^−9^ ± 0.1 ^b^

Different letters within the same column indicate statistically significant differences (*p* < 0.05).
